# Feline leukocyte immunophenotyping: an optimised whole-blood flow cytometry protocol

**DOI:** 10.1016/j.mex.2026.103869

**Published:** 2026-03-19

**Authors:** Rafael S. Lopes, Paulo Rodrigues-Santos, Maria dos Anjos Pires, Eduardo Costa, Ana Catarina Figueira, João F. Requicha, Pedro Carvalho

**Affiliations:** a Department of Veterinary Sciences, University of Trás-os-Montes e Alto Douro (UTAD), Quinta de Prados 5000-801 Vila Real, Portugal; b Vasco da Gama Research Center (CIVG), University School Vasco da Gama (EUVG), Campus Universitário de Lordemão, *Av*. José R. Sousa Fernandes 3020-210 Coimbra, Portugal; c Animal and Veterinary Research Center (CECAV) - Associate Laboratory for Animal and Veterinary Science (AL4Animals), UTAD, Vila Real, Portugal; d University of Coimbra, Faculty of Medicine (FMUC), Institute of Immunology, Center for Neurosciences and Cell Biology (CNC), Laboratory of Immunology and Oncology, Center for Investigation in Environment, Genetics and Oncobiology (CIMAGO), Coimbra Institute for Clinical and Biomedical Research (iCBR), Center for Innovation in Biomedicine and Biotechnology (CIBB), and Clinical and Academic Center of Coimbra (CACC), Coimbra, Portugal; e University of Coimbra, Institute of Experimental Pathology, Faculty of Medicine, Azinhaga de Santa Comba, 3000-548 Coimbra, Portugal; f CQC-IMS, Chemistry Department, University of Coimbra, Rua Larga 3004-535; g OneVet Group, Hospital Veterinário Universitário de Coimbra (HVUC), *Av*. José R. Sousa Fernandes, 297, 3020-210 Coimbra, Portugal; h Vetherapy, 479St, San Francisco, CA 94103, USA

**Keywords:** Flow cytometry, Feline leukocytes, Extracellular immunophenotyping, Antibody titration, MIFlowCyt

## Abstract

**Background:**

**:** Flow cytometry is a powerful tool for immunophenotyping, but its application in feline samples is challenging due to species-specific blood characteristics, a paucity of standardised protocols and high reagent costs. These limitations may compromise sample quality, antibody performance, and the reproducibility of the results.

**Methods:**

**:** An optimal protocol for extracellular immunophenotyping of feline leukocytes from peripheral whole-blood was developed, adapting established human flow cytometry methods. Key adaptations included improved blood collection process, assessing sample preservation, and titrating antibodies. Detailed MIFlowCyt-compliant information on cytometer configuration, compensation procedures, gating strategy, and controls is provided in Supplementary File S2.

**Results:**

**:** The enhanced blood collection significantly improved erythrocyte lysis rendering the samples more suitable for cytometric analysis. Baseline leukocytes viability, assessed by trypan blue exclusion was 98–100%. The use of a cellular antigen stabilisation reagent preserved feline peripheral whole-blood samples without detectable loss of surface antigen expression for up to 14 days. Antibody titration showed that most monoclonal antibodies were effective at 1.5 µL, reducing usage by up to 85%, while CD5 required 3 µL.

**Conclusion:**

**:** This improved, cost-effective and reproducible protocol address major technical limitations in feline flow cytometry and provides a practical framework for the reliable leukocyte immunophenotyping in clinical diagnostics, research, and comparative immunology.

## Graphical abstract

**Figure:**
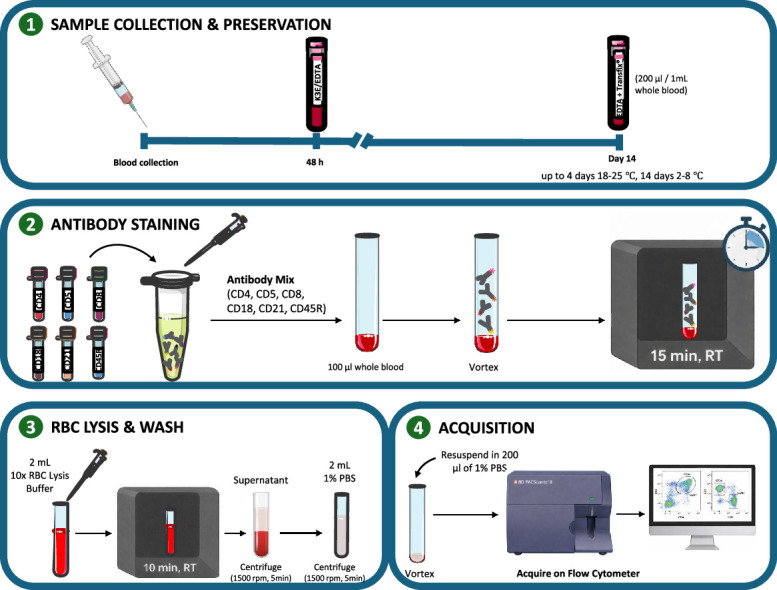

**Specifications table**

**Table:** 

**Subject area**	Veterinary Science and Veterinary Medicine
**More specific subject area**	Feline Haematology
**Name of your protocol**	Feline Leukocyte Cytometric Analysis Technique (FL-CAT)
**Reagents/tools**	All materials and equipment are listed in the appropriate article section
**Experimental design**	This study proposes an improved protocol for the immunophenotyping of feline leukocytes for detection by flow cytometry. The protocol has been refined and built upon the basis of existing human protocols. The development of these specific adaptations to cat samples was motivated by the objective of rendering them more user-friendly, economically less expensive, faster and more productive.
**Trial registration**	Not applicable
**Ethics**	This study protocol does not involve human subjects, animal experiments, or data collection from social media platforms, thus, informed consent is not required.
**Value of the Protocol**	**Standardisation and reproducibility:** The protocol provides a MIFlowCyt-compliant, standardised workflow specifically adapted to feline whole-blood samples. The integration of defined cytometric controls, gating strategies, compensation procedures, and viability assessment within the framework contributes to enhancing the reproducibility of results across different laboratories and facilitating consistent data analysis. **Logistical flexibility:** The integration of a cellular antigen stabilization reagent enables the extended preservation of feline whole-blood samples for delayed analysis, thereby supporting multicentric studies, remote sample shipment, and routine clinical workflows where immediate access to flow cytometry equipment is limited. **Economic sustainability:** Quantitative antibody titration leads to a significant reduction in monoclonal antibody consumption while preserving signal integrity, achieving up to an 85% reduction in reagent usage. This substantial reduction in operational costs and minimisation of reagent waste is a key factor in enabling the broader implementation of multiparametric flow cytometry in feline medicine. **Clinical and translations relevance**: The protocol provides a reliable immunophenotyping of feline leukocytes for clinical diagnostics, monitoring of immune-mediated and infectious diseases, and assessment of therapeutic responses. Furthermore, it provides a standardised framework for comparative and translational immunology studies involving feline models, extending their usefulness and making them highly relevant.

## Background

Flow cytometry is a gold-standard technique for analysing the qualitative and quantitative characteristics of individual whole cells and cellular components, as well as the cell cycle and DNA content [1].

Lymphocyte populations play a central role in the immune response and can be efficiently analysed using flow cytometry [2,3].

In veterinary medicine it is vital to understand the feline immune system, particularly in cases of immune-mediated and oncological diseases or response to infection [4,5]. However, feline whole-blood sample analysis remains particularly challenging due to the limited availability of standardised protocols, high reagent costs, and the distinct biological and pre-analytical characteristics of feline blood, including increased plasma protein content, marked platelet reactivity, and a pronounced tendency for erythrocyte aggregation, which collectively compromise red blood cell lysis efficiency, sample processability, and immunofluorescent signal stability.

Although flow cytometry is an efficient method for the characterisation of lymphocyte subsets, the majority of available feline studies rely on protocols that have been adapted from studies in other species [6]. Furthermore, these studies frequently focus on phenotypic characterisation rather than on operationally validated workflows. A recent study by Meneses-Nava et al. [7] provided an extensive immunophenotypic characterisation of feline lymphoid subsets using fresh whole-blood and a large multicolor antibody panel. While this work significantly advances biological knowledge of feline leukocytes populations, it relies on samples processed within 24 h and does not address pre-analytical variability, long-term sample preservation, or economic antibody optimisation [7].

These factors remain major logistical limitations for routine, remote, and multicentric cytometric workflows.

Furthermore, sample degradation occurring during storage and transportation imposes additional constraints on the execution of feline cytometric analysis, particularly in cases where access to the equipment necessary for such analysis is delayed. Concurrently, the significant volume of monoclonal antibodies (mAbs) recommended by manufacturers leads to a substantial increase in experimental costs, which limits the routine use of multiparametric immunophenotyping in feline medicine [8,9].

A critical need therefore remains for a validated, MIFlowCyt-compliant, cost-effective workflow specifically adapted to the biological and logistical particularities of feline blood. The present study addresses this gap by providing an improved protocol for the extracellular immunophenotyping of feline leukocytes. This protocol integrates improved blood collection and erythrocyte lysis strategies, extended sample preservation using a cellular antigen stabilization reagent, defined cytometric controls and gating strategies, and quantitative antibody titration to optimise reagent consumption while maintaining signal integrity.

The present protocol provides a standardised and reproducible framework for feline leukocyte immunophenotyping, thereby supporting clinical diagnostics, multicentric studies, and comparative immunology [10]. This protocol is designed to facilitate the broader and economically sustainable implementation of flow cytometry in feline medicine.

## Description of protocol

***Step 1 - Sample collection and preservation procedure***

*Materials*

• 2 ml sterile syringes
• 23 G, 25 mm sterile needles
• Aquisel® (Vaculab®, China) K3 EDTA 0.5/1.0 mL tubes
• Pipettes
• Micropipettes
• Compress cotton gauze
• Permanent marker

Reagents

• Transfix® (Cytomark, Buckingham, UK)
• Chlorhexidine
• Alcohol

Equipment

• Sample mixer (HulaMixer®, Life technologies™, USA)

Methods

1. Prepare the syringe and needle
2. Restrain and position the animal with minimal stress
3. Identify and aseptically prepare the jugular venipuncture site
4. Apply alcohol with a gauze pad followed by chlorohexidine
5. Perform the jugular venipuncture with precision, with minimal vacuum pressure to prevent turbulence and vein collapse
6. The whole blood sample should be placed in the collection tube with EDTA as soon as possible and gently shaken for one minute to ensure contact with the internal tube anticoagulant surfaces, ensuring good homogenisation
7. Samples are stored at room temperature (RT) in the sample mixer until analysis (up to 48 h)
8. In cases where the sample is to be processed over 48 h, Transfix® reagent should be added proportionally according to the manufacturer’s instructions (200 µl Transfix® for 1 ml of whole blood), carefully homogenised and stored at 18–25 °C up to 4 days or 2–8 °C up to 14 days.

Step 2 – Leukocytes extracellular staining

Materials

• EDTA Whole blood sample ± Transfix®
• 200 µl pipettes
• 100 µl pipettes
• 10 µl pipettes
• Flow cytometry tubes
• Permanent marker
• Cytometer tube rack

Reagents

• Monoclonal antibodies:
  ○ CD5 Anti-cat – clone FE1.1B11 (BIO-RAD®)
  ○ CD4 Anti-cat – clone vpg34 (BIO-RAD®)
  ○ CD8 Anti-cat alpha/beta purified – clone vpg9 (BIO-RAD®)
  ○ Rat Anti-Mouse IgG1 – clone X56 (BIO-RAD®)
  ○ CD18 Mouse Anti-Dog – clone CA1.4E9 (BIO-RAD®)
  ○ CD21 Mouse Anti-Dog – clone CA2.1D6 (BIO-RAD®)
  ○ CD45R Rat Anti-Mouse – clone RA3–6B2 (BIO-RAD®)
• 10x Red blood cells (RBC) lysis buffer solution (BD FACS™ lysing solution)
• PBS 1% solution

Equipment

• Countess ™ 3 (Thermo Fisher Scientific, USA)
• Freezer
• Dark incubation chamber
• Timer
• Vortex (MX-S®, China)
• Centrifuge (model 5810R, Eppendorf®, Germany)
• Flow cytometry analyser BD FACSCanto II (Becton Dickinson (BD), San Jose, USA)

Methods

1. Pipette 10 μL of whole blood into a microcentrifuge tube
2. Add 10 μL of trypan blue solution (0,4%) and gently mix
3. Load the mixture into the automated cell counter chamber and record the percentage of viable cells
4. Pipette 100 μL of whole blood to a cytometry tube
5. Add 10 μL of selected primary antibodies to the cytometry tube
6. Vortex and incubate for 15 min in the dark chamber at RT
7. Add 2 mL of 10x RBC BD FACS™ lysing solution
8. Vortex and incubate for 10 min in the dark chamber at RT
9. Centrifuge during 5 min at 1500 rpm without brake
10. Discard the supernatant
11. Add 2 mL of PBS 1% and vortex
12. Centrifuge during 5 min at 1500 rpm without brake
13. Discard the supernatant
14. Pipette 200 μL of PBS 1%
15. Vortex and acquire them on the flow cytometer

**Fig. 1:**
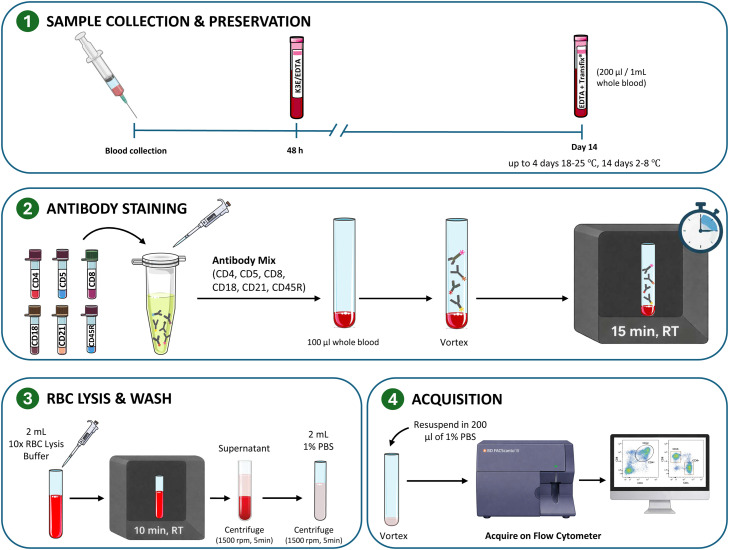
Schematic overview of the optimized workflow for extracellular immunophenotyping of feline leukocytes by flow cytometry.

Note: If secondary antibodies are employed, a wash with PBS 1% should be conducted after step 6, followed by centrifugation, supernatant discard and a further 15-minute incubation period in a dark chamber at RT with the secondary antibody after vortex Fig. 1.

### Collection technique for successful lysis

The methodology and quality of blood collection were found to have a direct impact on the processing and validity of the sample.

Cat blood contains a comparatively elevated concentration of plasma proteins, most notably fibrinogen and globulins, which promote the adhesion of erythrocytes. These proteins reduce the electrostatic repulsion between RBC, creating conditions that facilitate their aggregation [11]. In addition, feline platelets exhibit a high degree of reactivity, with a propensity to activate and form aggregates during the blood collection process, even in the absence of specific stimuli [12].

To obtain a high-quality sample, it is imperative to obtain blood by venipuncture from larger calibre vessels (jugular), employing syringes with a low volume capacity (no >2 mL) and needles with a calibre of 23 G or less. This method ensures that low vacuum pressure is generated, thereby preventing vessel collapse or blood turbulence within the syringe blood collection.

In this step, to minimise the risk of red blood cell aggregation, the collection procedure was adapted by placing a drop of EDTA anticoagulant in the needle hub.

The collected sample (without anticoagulant) should be placed in the EDTA tube, as soon as possible, and the sample should be gently homogenised to ensure that the blood contact with the entire inner wall of the collection sample tube.

This step is critical to ensure the integrity of the sample and the subsequent erythrocyte lysis process (point 5) that provided different challenges related to the sample collection and preparation.

In accordance with the established original protocol, observation of the erythrocyte lysis would only be attainable after the mAbs incubation. Another adaptation to the protocol that should be considered is the prior incubation of 100 µL whole blood samples for 15 min in a dark chamber at RT in 2 ml of the lysis solution, points 4, 5, 6 and 7 of step 2.

Following point 7 at step 2, the effectiveness of the lysis process can be evaluated by the absence of erythrocyte sediment at the bases of the cytometry tubes. This serves to validate both the sample for processing and the efficacy of the collection technique, as no sedimented or pellet formation was observed.

Once this evaluation has been completed, it is possible to select the samples that can be processed and successfully analysed in the flow cytometer promoting reagent and cost savings in non-lysed samples.

### Sample viability and preservation over time

Following the acquisition of the sample, it is mandatory that the cells are processed within 48 h to ensure their viability and the validity of the results. In circumstances where the cytometry equipment is not available or is located at a considerable distance, it is crucial to ensure the sample preservation until they can be analysed. This approach is equally applicable to samples collected in multicentric studies that are not delivered to the laboratory within 48 h.

Independent assessment of cell viability by trypan blue exclusion demonstrated high baseline leukocyte viability (98–100%) in fresh whole-blood samples, confirming that pre-analytical handling did not compromise cell integrity (Supplementary File S3). Trypan blue exclusion was used to assess baseline cell integrity prior to staining and acquisition. While this method is appropriate for the evaluation of gross membrane integrity, it differs from flow cytometry viability dyes, which assess membrane permeability at the time of acquisition and enable the exclusion of non-viable events during analysis.

To mitigate the effects of time on samples degradation, the use of a cellular antigen stabilisation reagent was tested (Transfix®, Cytomark, UK). According to the manufacturer's specifications (for human blood samples), the reagent demonstrates a preservative effect when the ratio of reagent to blood sample is 200 µL: 1 mL at a temperature range of 2–8 °C, and an expiration time period up to 14 days.

An experiment was conducted to determine the similarity of the Transfix® capacity on cat blood samples. The results demonstrated that no differences were observed between fresh and Transfix®-preserved feline blood, nor at different time points of the sample preserved for up to 14 days after collection.

### Antibody optimisation and validation

One of the major costs of this protocol relates to the number and specificity of antibodies used.

In order to verify and corroborate the manufacturer's data sheet on antibody specificity, a confirmatory procedure was conducted. Immunoassays were employed to assess both the specificity and efficacy of the antibodies (Supplementary File 2).

According to the data sheet, the manufacturer recommends using 10 µL of the suggested antibodies working dilution to label 10^6^ cells in 100 µL of whole-blood sample.

Due to financial constraints and aiming to economise antibody consumption, a titration test was performed to find the minimum volume required without affecting the labelling and visualisation of fluorescence cytometric analysis.

In this experiment, the same sample was titrated with 5 µL, 3 µL and 1.5 µL of mAbs.

**Fig. 2:**
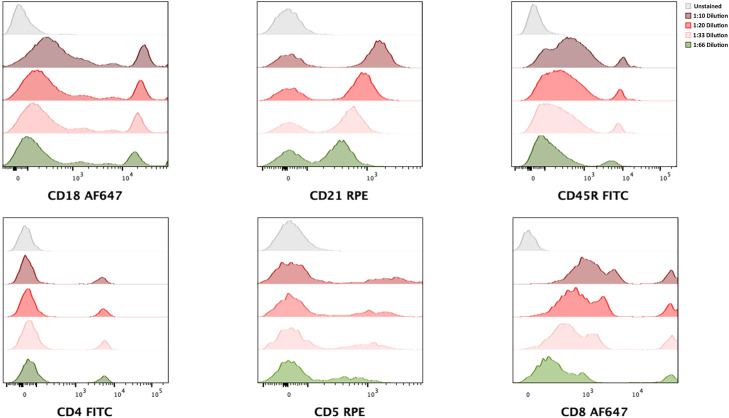
Representative leukocyte-gated histograms illustrating antibody titration for feline leukocyte immunophenotyping. All histograms display singlet leukocytes, defined by FSC-A versus SSC-A morphological gating, followed by FSC—H versus FSC-A singlet discrimination. Titration was performed for CD18, CD21, CD45R, CD4, CD5 and CD8 monoclonal antibodies using three antibody volumes: 10 µL (1:10 dilution), 5.0 µL (1:20 dilution), 3.0 µL (1:33 dilution) and 1.5 µL (1:66 dilution). Minimal working volumes were selected based on optimal signal-to-noise ratios.

The cluster of differentiation 4 (CD4), CD8, CD18, CD21 and CD45R biomarkers, showed similar labelling and fluorescence between the 3 vol of used mAbs. The only exception was CD5, where optimal fluorescence was obtained with a minimum of 3 µL (Fig. 2). Quantitative assessment of antibody titration was performed using stain index values calculated with the StainIndex plugin in FlowJo v10.10.0, and the results are reported in Supplementary Table S1.

The original protocol is available as Supplementary File S1.

### Software

FlowJo™ 10.10.0 version; Microsoft® Excel 2019, 16.78 version; Microsoft Corp., USA and GraphPad Prism 10.4.2 version software for data display and analysis

## Protocol validation

The use of flow cytometry in feline medicine is still largely dependent on protocols adapted from human or canine workflows. Such protocols are often lacking formal validation, standardized gating strategies, and defined pre-analytical controls. Despite recent studies having expanded the biological characterisation of feline lymphocyte subsets using multicolor panels and fresh blood samples, there remain limitations. These are due to the requirement for immediate processing and the absence of validated approaches addressing pre-analytical variability, long-term preservation, and reagent optimisation.

Standardisation of cytometric acquisition and analysis was performed in accordance with the recommendations of MIFlowCyt. A description of the detailed gating strategy, cytometric controls, compensation procedures and cytometer configurations is provided in the Supplementary File S2.

The potential CD4/CD8 double-positive events were not interpreted as a distinct biological population. In consideration of the recognised constraints associated with extracellular staining and fluorochrome spillover, such occurrences were regarded as potentially artefactual and were excluded from subsequent analyses. Consequently, the protocol has been optimised for the reliable identification of single-positive CD4+ and CD8+ *T*-cell populations, as opposed to the discrimination of true double-positive subsets.

The present protocol was validated to address these limitations through targeted refinement of sample collection, preservation, and laboratory processing. Optimised jugular venipuncture with low-turbulence blood collection significantly improved erythrocyte lysis efficiency and sample acceptance for cytometric analysis. Secondary lysis attempts and temperature modifications (blood and lysis buffer at 37 °C) did not further improve lysis efficiency.

The necessity of processing samples within 48 h to maintain viability is challenging, particularly when the cytometry equipment is unavailable or located at distance. This limitation is critical for multicentric studies or samples requiring transportation. The use of a cellular antigen stabilization reagent (TransFix®) has been demonstrated to maintain the integrity of the leukocyte population and surface antigen expression for a period of up to 14 days, thereby facilitating delayed analysis and multicentric workflows [13,14], not only in human blood, but also in cat blood samples.

The elevated cost and consumption of mAbs represent a substantial financial constraint in feline flow cytometry. However, these limitations can be mitigated through targeted protocol refinements that enable the establishment of a robust, cost-effective, and species-adapted workflow.

Titration is a method of determining the required antibody concentration for the purpose of processing the highest signal of the positive and the lowest signal of the negative population. It is the most effective approach to eliminating nonspecific antibody binding [15].

Quantitative antibody titration demonstrates minimal volume of 1.5 µL comparable to higher antibody volumes for CD4, CD8, CD18, CD21 and CD45R, achieving up to 85% reduction in antibody consumption (6.6 times lower volume), while CD5 required 3.0 µL to maintain optimal signal, corresponding to 70% reduction (3.3 times lower volume), without loss of signal integrity.

**Fig. 3:**
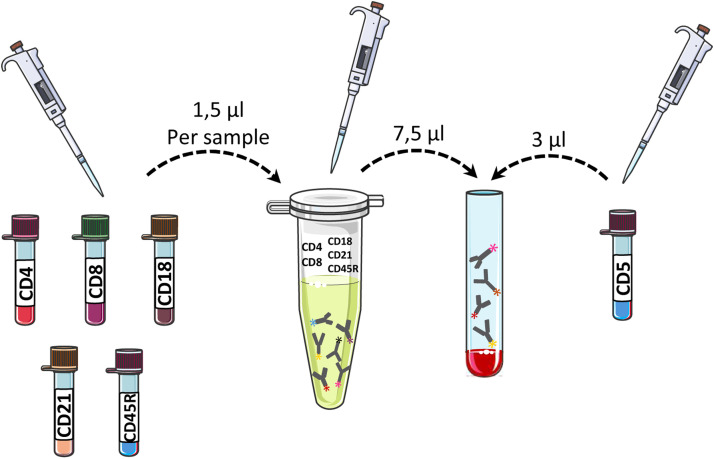
Schematic representation of antibody pre-mixing for feline leukocyte immunophenotyping. Minimal working antibody volumes (1.5 µL per marker and 3.0 µL for CD5) were pre-mixed in a single sterile microcentrifuge tube according to the number of samples to be processed. The total antibody cocktail volume was adjusted proportionally to the sample number, enabling reduced reagent consumption, minimised manual handling, and improved workflow reproducibility.

The pre-mixing of antibody cocktails has been demonstrated to reduce the consumption of disposable materials, manual handling, minimise ambient exposure of reagents, and improve workflow reproducibility (Fig. 3). The total volume was adjusted according to the number of samples to be processed.

Collectively, these validations demonstrate that the proposed workflow provides a robust, reproducible, and economically sustainable framework for feline leukocyte immunophenotyping.

## Limitations

Although the present protocol provides a standardised and MIFlowCyt-compliant workflow for feline leukocyte immunophenotyping, it is important to note that there are several limitations that must be considered. The validation was performed on a limited number of peripheral blood samples, and a broader multicentric evaluation would further strengthen the generalizability of the workflow. Furthermore, while the maintenance of immunophenotypic stability was ensured by extended sample preservation using a cellular antigen stabilization reagent, the potential effects on intracellular markers were not assessed and should be addressed in future studies.

Furthermore, the protocol is exclusively focused on extracellular immunophenotyping of leukocytes, excluding both intracellular staining procedures and functional assays. Despite substantial reduction in antibody consumption, access to species-reactive monoclonal antibodies remains limited in feline medicine, which may restrict the expansion of multiparametric panels.

Finally, although improvements in blood collection and erythrocyte lysis increased sample acceptance rates, rare cases of incomplete erythrocyte lysis may still occur, potentially affecting sample processability for cytometric analysis. It is recommended that future studies explore the development of additional strategies with a view to further minimising lysis failure and expanding the range of validated feline-specific reagents.

## CRediT author statement

RL: Conceptualization, Data curation, Formal Analysis, Investigation, Methodology, Writing– original draft, Writing– review & editing. PR-S: Conceptualization, Formal Analysis, Validation, Visualization, Writing– review & editing, Investigation, Methodology, Resources, Supervision. MP: Conceptualization, Formal Analysis, Supervision, Validation, Visualization, Writing – review & editing, Methodology. EC: Data curation, Formal Analysis. ACF: Conceptualization, Investigation, Methodology, Resources, Supervision. JR: Conceptualization, Data curation, Formal Analysis, Funding acquisition, Investigation, Methodology, Resources, Supervision, Validation, Visualization, Writing– original draft, Writing – review & editing. PC: Conceptualization, Formal Analysis, Supervision, Visualization, Writing– review & editing.

## Related research article

Adapted from human BD Biosciences protocol: Direct Immunofluorescence Staining of Whole-Blood Using a Lyse/No-Wash Procedure.

## For a published article

None.

Supplementary material

Supplementary File S1 - Original protocol for extracellular immunophenotyping.

Supplementary File S2 - Contour plots of cytometric analysis and gating strategy, controls, compensation procedures and detailed cytometer settings.

Supplementary File S3 - Baseline viability of feline whole blood assessed by trypan blue exclusion using automated cell counter.

Supplementary Table S1 - Antibody titration expressed as stain index (SI) values for feline leukocyte immunophenotyping, calculated using the StainIndex plug-in FlowJo Software v10.10.0.

## Declaration of competing interest

The authors declare that they have no known competing financial interests or personal relationships that could have appeared to influence the work reported in this paper.

## Data Availability

The data that has been used is confidential.

## Appendix Supplementary materials

Image, application 1
