# Supplementary material for: Feline leukocyte immunophenotyping: an optimised whole-blood flow cytometry protocol
Source: MethodsX. 2026 Mar 19;16:103869. doi: 10.1016/j.mex.2026.103869 (PMC13049955; doi:10.1016/j.mex.2026.103869)
Supplement: Supplementary file 1 [file mmc1.zip › mmc1.docx]

**Supplementary File S1 -** Original step-by-step protocol for extracellular immunophenotyping**.**

***Step 1 - Sample collection and preservation procedure***

*Materials*

- Sterile syringes
- Sterile needles
- Pipette A-2 without rubber (Aquisel^®^, Vaculab^®^, China)
- K3 EDTA 10 mL tubes
- Compress cotton gauze
- Tourniquet
- Permanent marker

*Reagents*

- Alcohol

*Equipment*

- Sample mixer (HulaMixer®, Life Technologies™, USA)

*Methods*

1. Prepare the patient ensuring the comfort in supine or low-recumbent position
2. Prepare the syringe and needle
3. Identify and aseptically prepare the venipuncture site with antiseptic, such 70% ethyl alcohol
4. Perform the venipuncture with precision at 15–30° angle
5. Collect the blood by slowly pulling back on the syringe plunger
6. Remove the tourniquet and gently withdraw the needle
7. Apply gentle pressure to the site with a clean gauze or cotton
8. Transfer the blood to the collection tube with K3 EDTA as soon as possible and gently mix for one minute (or 5-8 times) to ensure contact with the internal tube anticoagulant surfaces, ensuring good homogenisation
9. Store the samples in the sample mixer at room temperature until analysis

***Step 2 - Lymphocytes extracellular immunolabelling***

*Materials*

- EDTA whole blood sample
- 200 µL pipettes (Eppendorf^®^)
- 100 µL pipettes (Eppendorf^®^)
- 10 µL pipettes (Eppendorf^®^)
- Flow cytometry tubes
- Permanent marker
- Cytometer tube rack

*Reagents*

- Monoclonal antibodies (mAbs)
- 10x Red blood cells (RBC) lysis buffer solution
- Phosphate-buffered saline (PBS) 1%

*Equipment*

- Freezer
- Room temperature dark incubation chamber
- Timer
- Vortex (MX-S^®^, China)
- Centrifuge (model 5810R, Eppendorf^®^, Germany)
- Flow cytometry analyser BD FACSCanto II (Becton Dickinson (BD), San Jose, USA)

*Methods*

1. Pipette 100 μL of whole blood to a cytometry tube
2. Prepare the antibodies, vortex and spin down for 5 seconds
3. Add 10 μL of each antibody to the cytometry tube
4. Vortex and incubate for 15 minutes in the dark chamber at room temperature
5. Add 2 mL of 10x RBC lysis buffer solution
6. Vortex and incubate for 10 minutes in the dark chamber at room temperature
7. Centrifuge during 5 minutes at 1500 rpm without brake
8. Discard the supernatant
9. Add 2 mL of PBS 1% solution and vortex
10. Centrifuge during 5 minutes at 1500 rpm without brake
11. Discard the supernatant
12. Pipette 200 μL of PBS 1%
13. Vortex and acquire the tubes in the flow cytometer
