# Supplementary material for: Feline leukocyte immunophenotyping: an optimised whole-blood flow cytometry protocol
Source: MethodsX. 2026 Mar 19;16:103869. doi: 10.1016/j.mex.2026.103869 (PMC13049955; doi:10.1016/j.mex.2026.103869)
Supplement: Supplementary file 1 [file mmc1.zip › mmc2.docx]

**Supplementary File S2 –** MIFlowCyt-compliant cytometric configuration, gating strategy **(A)**, controls **(B)**, compensation procedures **(C)** and detailed cytometer settings **(D)** for feline lymphocyte immunophenotyping.

**(A) Gating strategy** – Representative contour plots illustrating the sequential gating strategy used for immunophenotyping of feline peripheral blood lymphocytes. Singlet discrimination was first performed using FSC-H versus FSC-A to exclude doublets (Plot **A1**; gate: all events). Events were then gated based on forward scatter (FSC-A) and side scatter (SSC-A) properties to define a lymphocyte-enriched region according to cell size and granularity (Plot **A2**; parent gate: singlets). Within this region, an operational leukocyte-enriched gate was defined using CD45R and CD18 expression to exclude residual debris and non-lymphocyte events (Plot **A3**; parent gate: lymphocyte-enriched region). Within the leukocyte-enriched lymphocyte population, B cells were identified as CD21⁺ events with supportive CD45R staining (Plot **A4**; parent gate: CD45R⁺CD18⁺ lymphocytes). T cells were defined by CD5 expression within the lymphocyte gate (Plot **A5**; parent gate: lymphocyte-enriched region). Finally, CD5⁺ T cells were further subdivided into CD4⁺, CD8⁺, and CD8^lo^ T-cell subsets based on CD4 and CD8 expression (Plot **A6**; parent gate: CD5⁺ T cells).

**Gate on lymphocyte-enriched region cells**

**A3 -** CD45R vs CD18 showing identification of the CD45R^+^ CD18^+^ lymphocyte population within the lymphocyte-enriched gate.


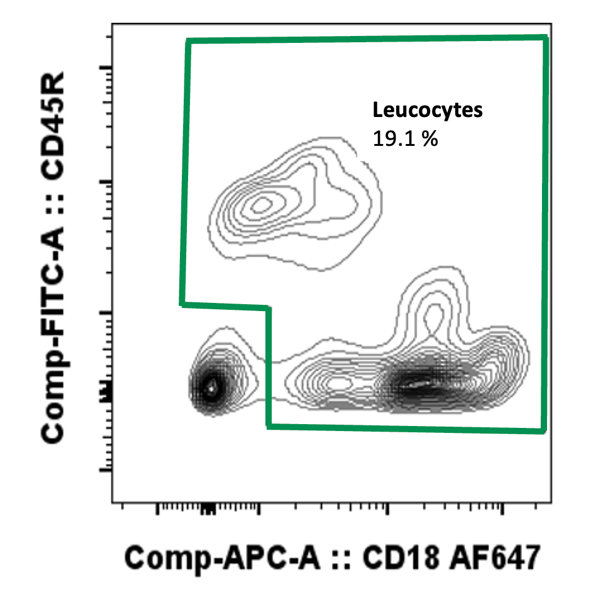


**A2 -** FSC-A vs SSC-A showing morphological identification of the lymphocyte-enriched region following singlet gating.


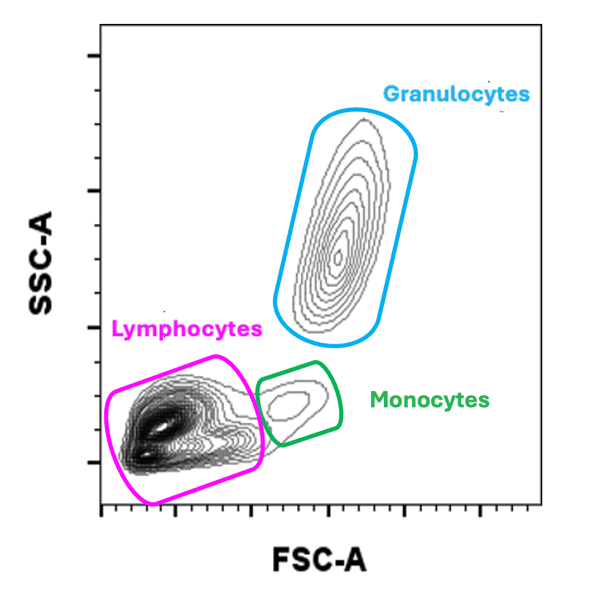


**Gate on single cells**

**A1 -** FSC-A vs FSC-H showing singlet discrimination to exclude doublets (gate: all events).


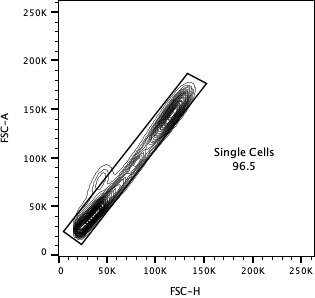

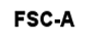


**FSC-H**

**Single cells**

96.5%


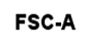


**Gate on all events**


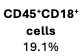


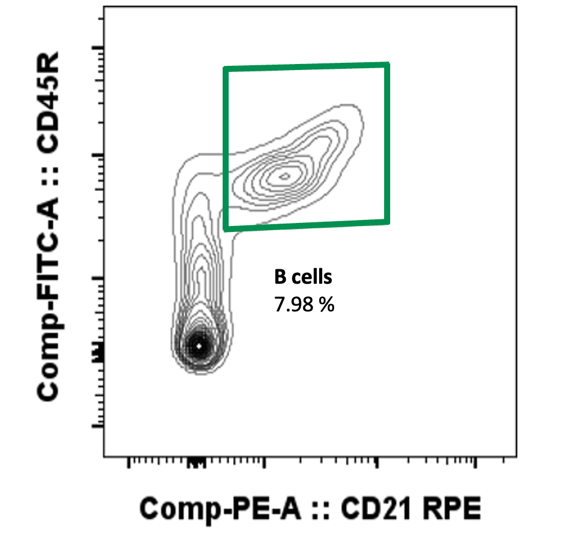


**Gate on CD45R^+^CD18^+^ lymphocytes**

**A4 –** CD45R vs CD21 plot showing identification of the CD45R^+^ CD21^+^ B-cell population.

**Gate on lymphocyte-enriched region cells**

**A5 –** CD5^+^ vs SSC-A plot showing identification of the CD5^+^ T-cell population within the lymphocyte-enriched gate.


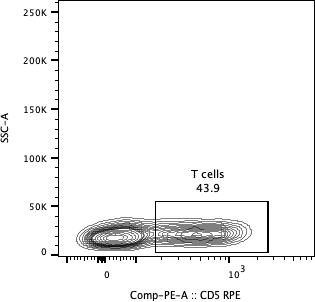

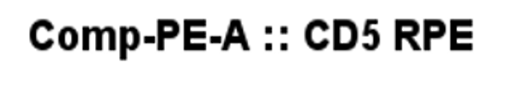

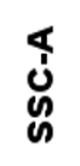


**T cells**

43.9%


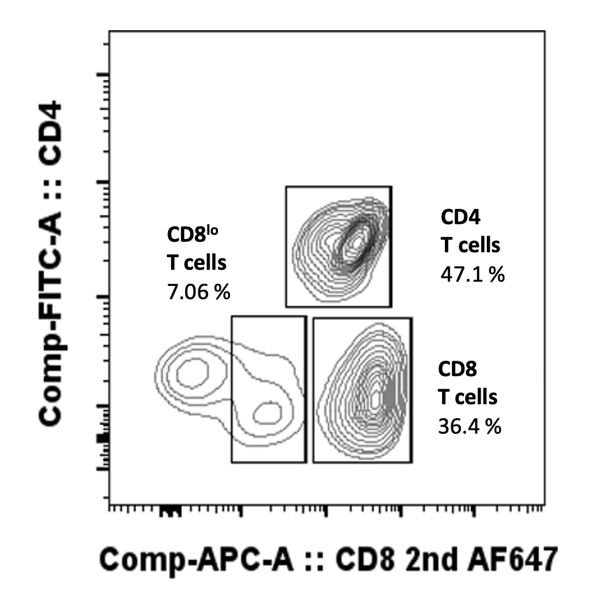


**Gate on T cells**

**A6 –** CD4 vs CD8 plot showing discrimination of CD4^+^ and CD8^+^ T-cell subsets within the CD5^+^ T-cell gate.

**T cells**

43.9%

**(B) Cytometric controls** - Summary of fluorescence channels, detector parameters, and photomultiplier tube (PMT) voltages applied for all analyses. Unstained, fluorescence minus one (FMO), and isotype controls were used to define gating thresholds.

| **Parameters** | **Type** | **Voltage** |
| --- | --- | --- |
| FSC | A, H, W | 324 |
| SSC | A, H, W | 395 |
| FITC | A | 445 |
| PE | A | 434 |
| APC | A | 562 |

**(C) Compensation procedures** - Compensation matrix indicating fluorochrome spectral overlap and percentage spillover values applied for FITC, PE, and APC channels.

| **Fluorochrome** | **- % Fluorochrome** | **Spectral Overlap** |
| --- | --- | --- |
| PE | FITC | 22.65 |
| APC | FITC | 0.11 |
| FITC | PE | 1.06 |
| APC | PE | 0.07 |
| FITC | APC | 0.00 |
| PE | APC | 0.03 |

**(D) Instrument configurations** - BD FACS Canto II cytometer configuration and optical filter settings (BD FACSDiva software version 6.1.3) used for all acquisitions.

| **Blue** | SSC | 488/10 |  |
| --- | --- | --- | --- |
|  | FITC | 530/30 | 502 LP |
|  | PE | 585/42 | 556 LP |
| **Red** | APC | 660/20 |  |
