# Supplementary material for: Feline leukocyte immunophenotyping: an optimised whole-blood flow cytometry protocol
Source: MethodsX. 2026 Mar 19;16:103869. doi: 10.1016/j.mex.2026.103869 (PMC13049955; doi:10.1016/j.mex.2026.103869)

**Supplementary File S3 –** Baseline viability of feline whole blood assessed by trypan blue exclusion using an automated cell counter (Countess™ 3, Thermo Fisher Scientific), demonstrated 100% viable cells, with no detectable dead cells and no aggregation.


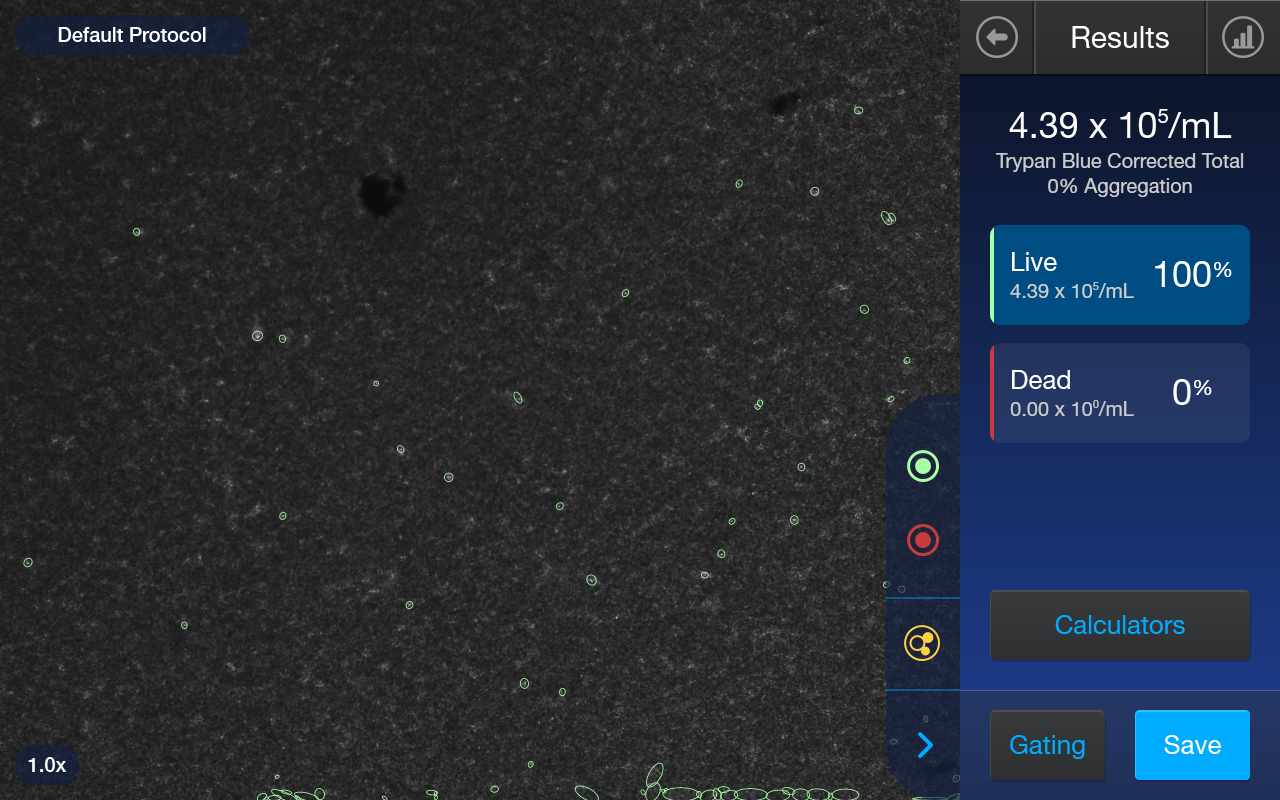

Supplement: Supplementary file 1 [file mmc1.zip › mmc3.docx]
